# Research Trends and Hotspot Analysis of Conjunctival Bacteria Based on CiteSpace Software

**DOI:** 10.1155/2020/2580795

**Published:** 2020-10-05

**Authors:** Zhenyu Wang, Chen Huang, Xuemin Li

**Affiliations:** ^1^Department of Ophthalmology, Peking University Third Hospital, Beijing, China; ^2^Beijing Key Laboratory of Restoration of Damaged Ocular Nerve, Peking University Third Hospital, Beijing, China; ^3^Medical Research Center, Peking University Third Hospital, Beijing, China

## Abstract

**Objective:**

To sort out the literature related to conjunctival bacteria and summarize research hotspots and trends of this field.

**Materials and Methods:**

The relevant literature data from 1900 to 2019 was retrieved from the Web of Science Core Collection database. After manual selection, each document record includes title, author, keywords, abstract, year, organization, and citation. We imported the downloaded data into CiteSpace V (version 5.5R2) to draw the knowledge map and conduct cooperative network analysis, discipline and journal analysis, cluster analysis, and burst keyword analysis.

**Results:**

After manual screening, there were 285 relevant papers published in the last 28 years (from 1991 to 2019), and the number is increasing year by year. The publications of conjunctival bacteria were dedicated by 1381 authors of 451 institutions in 56 countries/regions. The United States dominates this field (82 literatures), followed by Germany (23 literatures) and Japan (23 literatures). Overall, most cited papers were published with a focus on molecular biology, genetics, nursing, and toxicology. Most papers fall into the category of ophthalmology, veterinary sciences, and pharmacology and pharmacy. The only organized cluster is the “postantibiotic effect,” and the top 5 keywords with the strongest citation bursts include “postoperative endophthalmiti(s),” “infectious keratoconjunctiviti(s),” “conjunctiviti(s),” “resistance,” and “diversity”.

**Conclusion:**

The global field of conjunctival bacteria has expanded in the last 28 years. The United States contributes most. However, there are little cooperation among authors and institutions. Overall, this bibliometric study organized one cluster, “postantibiotic effect”, and identified the top 5 hotspots in conjunctival bacteria research: “postoperative endophthalmiti(s),” “infectious keratoconjunctiviti(s),” “conjunctiviti(s),” “resistance,” and “diversity”. Thus, further research focuses on these topics that may be more helpful to prevent ocular infection and improve prophylaxis strategies to bring a benefit to patients in the near future.

## 1. Introduction

Resident bacteria flora can be detected in conjunctiva and conjunctival sac of healthy people, and the positive rate of conjunctival sac bacteria culture is about 20% [1–3]. It is generally believed that these resident bacteria in the conjunctiva and conjunctival sac will not cause disease. However, according to the results of several studies, these resident bacteria may cause opportunistic infection on patients with immune deficiency. In some serious cases, they may cause endophthalmitis, leading to irreversible severe visual impairment in a short period of time [4, 5]. Up to now, the treatment and prevention of conjunctival bacterial infection are mainly based on the use of antibiotics. However, with the extensive use of antibiotics, the resistance of bacteria towards antibiotics in conjunctiva and conjunctival sac is increasing [6, 7]. It is necessary to summarize literature in order to further understand the research hotspot of bacterial flora in conjunctiva and conjunctival sac and to provide research direction and basis for subsequent targeted research.

Bibliometrics is a subject that uses mathematical and statistical methods to conduct quantitative analysis based on the change of the number of papers, authors, keywords, and other measurement objects over time. It has been widely utilized to evaluate the importance and summarize hotspots of scientific studies in various fields, including medicine, ecology, and geology [8–11]. For instance, Shi et al. [12] predicted the clinical treatment of atrial fibrillation as an important research frontier by conducting bibliometric analysis on relevant research from 2004 to 2018. Qin et al. [13] summarized the research focuses of cerebral ischemia-reperfusion with application of CiteSpace and VOSviewer. Zou et al. [14] conducted bibliometric analysis on oncolytic virus research from 2000 to 2018, summarizing the top 4 hotspots of oncolytic virus research. Lu et al. [15] conducted scientometric analysis of SIRT6 studies, pointing out that there has been little analysis of how SIRT6 effects are part of more complex systems.

Also, there are a few bibliometric studies that focus on ophthalmologic diseases. For example, in the process of research on dry eye, Boudry et al. used VOSviewer to conduct bibliometric analysis on the literature related to dry eye in the web of science database and finally obtained the research trends and hot spots related to dry eye [16]. Schargus et al. analyzed the articles related to dry eye published in the Institute of Scientific Information database from 1990 to 2016 and summarized the articles cited most frequently [17]. Ramin et al. used Histcite software to analyze glaucoma published in the past 20 years [18]. Caglar et al. analyzed the relevant literature of diabetic retinopathy published in 1980-2014 and summarized the pathogenesis of diabetic retinopathy [19]. Zhao et al. [20] utilized CiteSpace V for analyzing and displaying the research trends of conjunctivochalasis. Among all the knowledge visualization software used in bibliometric analysis, CiteSpace is widely used for its powerful function of document sorting and mapping [21]. It was invented by professor Chen et al. in 2004 [21].

In this study, CiteSpace is used to sort out the literature related to conjunctival bacteria in the Web of Science Core Collection database. Besides, scientific metrology, data visualization, and statistical analysis methods (including citation analysis, cocitation analysis, cluster analysis, and data visualization analysis) are used to analyze the literature related to conjunctival bacteria from 1991 to 2019 and draw the knowledge map, summarizing research hotspots and trends in this field.

## 2. Materials and Methods

### 2.1. Data Acquisitions

The literature data was retrieved from the Web of Science Core Collection database using advanced search strategy. The key topic for retrieval is TS = (Conjunctivas OR “Palpebral Conjunctiva” OR “Conjunctiva, Palpebral” OR “Bulbar Conjunctiva” OR “Conjunctiva, Bulbar”) AND TS = (bacteria OR eubacteria). The refining method is (1) document types (article or review); (2) languages (English); (3) timespan: 1900-2019; (4) index: SCI-expanded, SSCI, a & HCI, cpci-s, CPCI-SSH, esci, ccr-expanded, IC. A total of 290 relevant literature records were retrieved. We excluded the studies in which human conjunctiva was not the subject. And then, we got the final literature database set. Each document record includes title, author, keywords, abstract, year, organization, citation, and other relevant information. Since the data were retrieved from a public open-accessed database, it did not involve ethical issues.

### 2.2. Analyzing Tools and Statistical Methods

We imported the downloaded data into CiteSpace V (version 5.5R2) for further analysis. The elementary parameter settings of the CiteSpace software were set as follows: (1) time slicing: from 1991 to 2019; years per slice: 5; (2) term source: title, abstract, author keywords, keywords plus; (3) selection criteria: TOP N%: we set different TOP N% for different node types. For country, institution, and source, the TOP N% we set was 100.0%. While for authors, we select top 5.0% of most cited or occurred items from each slice. (4) Pruning: no pruning; (5) visualization: cluster view-static and show merged network.

By running the software, we drew knowledge maps and conducted cooperative network analysis. Discipline and journal analysis were conducted using dual-mapping analysis (dual-map overlay). Cluster analysis and burst keywords analysis are used to study the research trends and hotspots in the field of conjunctival bacteria. The map of distribution of countries was drawn using Excel 2019.

## 3. Results

### 3.1. Published Outcomes and Cited Outcomes

**Figure 1:**
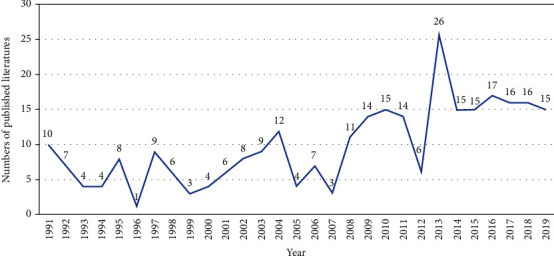
Total number of published literatures on conjunctival bacteria over time.

**Figure 2:**
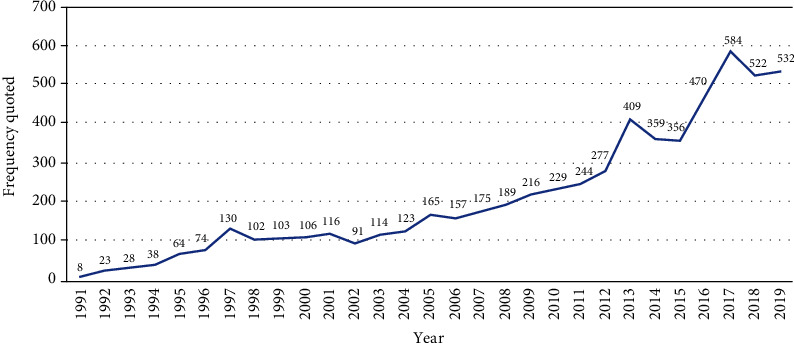
Frequency quoted on conjunctival bacteria over time.

After manual screening, there were 285 published papers related to conjunctival bacteria from 1991 to 2019. As shown in Figure 1, the number of papers on conjunctival bacteria was relatively small from 1991 to 2007, with 10 or less papers published annually except for 12 papers published in 2004. Since 2008, with the continuous deepening of the research on conjunctival bacteria by researchers all over the world, the number of papers published has steadily increased. In addition to 6 papers published in 2012, the number of papers published in other years is more than 10 annually, of which the highest number is 26 in 2013. In terms of the number of citations in the Web of Science Core Collection database, as shown in Figure 2, the citation frequency of such literature has generally increased since the first publication of literature related to conjunctival bacteria in 1991, reaching the peak in 2017 with a total of 584 citations.

### 3.2. Results of Cooperation

#### 3.2.1. Distribution of Countries/Regions

According to the results, the total number of documents retrieved from the Web of Science Core Collection database is small, and the number of papers published by a large number of countries, regions, institutions, and authors is similar and less than 3. In order to optimize the analysis strategy and improve the analysis efficiency, we set the “years per slice” to 5 and set different TOP N% for different node types. For country, the TOP N% we set was 100.0%.

**Figure 3:**
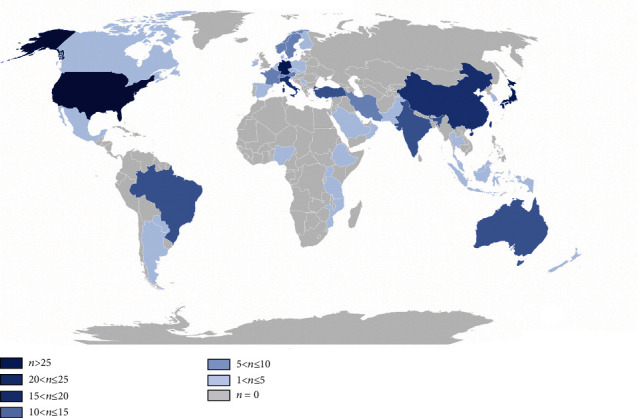
Global distribution map of the number of published literatures on conjunctival bacteria.

**Figure 4:**
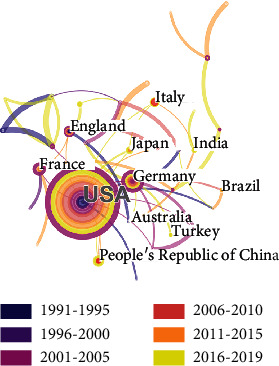
Cooperation network map of countries/regions on conjunctival bacteria. The size of each node represents the number of publications of each country/region. The thickness of the links between nodes indicates the close degree of the cooperative relationship between countries/regions.

After running the software, we gain the number of publications and the list of countries/regions where the literature related to conjunctival bacteria was published. In order to show the global distribution of literature related to conjunctival sac bacteria more directly, we input the data into Excel and draw Figure 3. As shown in Figure 3, we divided the countries/regions into 7 groups according to the number of publications (*n* > 25; 20 < *n* ≤ 25; 15 < *n* ≤ 20; 10 < *n* ≤ 15; 5 < *n* ≤ 10; 1 < *n* ≤ 5; *n* = 0). There is 1 country in the group 1 (*n* > 25), 2 countries/regions in the group 2 (20 < *n* ≤ 25), 3 countries/regions in the group 3 (15 < *n* ≤ 20), 4 countries/regions in the group 4 (10 < *n* ≤ 15), 6 countries/regions in the group 5 (5 < *n* ≤ 10), and 34 countries/regions in the group 6 (1 < *n* ≤ 5).  Table 1 shows the top 10 countries that published literature on conjunctival bacteria from 1991 to 2019, including the United States (82 papers), Germany (23 papers), Japan (23 papers), China (18 papers), and Italy (18 papers). In addition to these results, the CiteSpace software also provides the calculation results using knowledge maps.  Figure 4 shows the distribution of literature related to conjunctival sac bacteria and the cooperation network among countries and regions. Each node represents one country/region with published literature related to conjunctival sac bacteria, and the diameter of each node means the number of publications. Countries/regions with 10 published papers or more are labeled. The thickness of the links between nodes indicates the close degree of the cooperative relationship between countries/regions. According to Figure 4, there are 285 papers published in 50 countries/regions (50 nodes shown in Figure 4), of which the United States published the largest number of papers (82 papers). There are 52 links in total between nodes. Among them, the nodes connected with the United States are the most, with a total of 12 connections, respectively, connecting to Argentina, Italy, Paraguay, Japan, China, Germany, Gambia, Canada, England, Brazil, Israel, and France.

#### 3.2.2. Distribution of Institution

**Figure 5:**
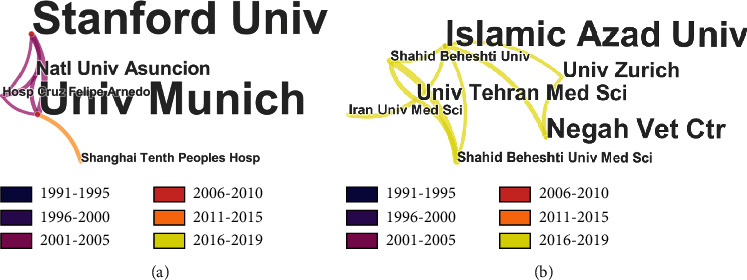
Partial cooperation network map of institutions on conjunctival bacteria. The size of each node represents the number of publications of each institution. The thickness of the links between nodes indicate the close degree of the cooperative relationship between institutions. (a) Cooperation groups among the institutions with the largest number of published literatures. (b) Cooperation groups among the institutions with the latest published literatures.

For institution, the TOP N% we set was also 100.0%. After running the software, we obtained the institution distribution and numbers of SCI articles related to conjunctival bacteria published by each institution in 1991-2019.  Table 1 shows the top 11 institutions that published literature on conjunctival bacteria between 1991 and 2019. According to the results, the University of Munich and the Stanford University are the institutions with the most conjunctival bacteria-related SCI publications. Both of them have 12 papers published. In addition, according to the calculation results of the CiteSpace, the publications of conjunctival bacteria were dedicated by 451 institutions. There is also a cooperative relationship among various institutions in the research of conjunctival bacteria. However, there are few nodes connected (only 554 links) compared with the cooperation at the national level. As shown in Figure 5, there is a cooperative relationship between the University of Munich and the Stanford University, the 2 institutions with the largest number of publications. Natl Univ Asuncion, Hosp Cruz Felipe Arnedo, and Shanghai Tenth Peoples Hosp are also partners of these two institutions. Most of the published papers were from 2001 to 2005. Besides, there are also cooperative relationships among several organizations that publish the latest literature. These institutions include Islamic Azad Univ (5 papers), Negah Vet Ctr (3 papers), Univ Tehran Med Sci (2 papers), Univ Zurich (2 papers), Iran Univ Med Sci (1 paper), Shahid Beheshti Univ (1 paper), and Shahid Beheshti Univ Med Sci (1 paper).

#### 3.2.3. Distribution of Authors

**Figure 6:**
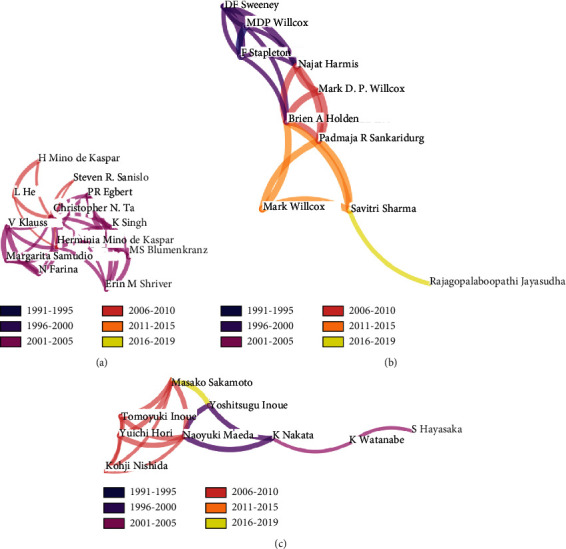
Partial cooperation network map of groups of authors with publications on conjunctival bacteria. (a) Partial cooperation network map of the group of authors with the largest publications on conjunctival bacteria. (b) Partial cooperation network map of the group of authors with the second largest publications on conjunctival bacteria. (c) Partial cooperation network map of the group of authors with the third largest publications on conjunctival bacteria.

According to our previous statistical analysis, a large number of authors only published a small number of papers each. With the exception of Christopher N. Ta (12 papers) and Herminia Mino de Kaspar (10 papers), the number of published papers by other authors is less than 5 each. There are 3 authors with 4 papers each, 19 authors with 3 papers each, 100 authors with 2 papers each, and 1257 authors with 1 paper each. Thus, we selected top 5.0% of the most cited or occurred items from each slice, and the maximum number of selected items per slice is 600. After running the CiteSpace software, 1381 nodes and 4799 links have been contained in the map of distribution of authors. The cooperation groups of authors are relatively independent.  Figure 6 shows the top 3 cooperation groups of authors with the largest publications on conjunctival bacteria. As shown in Figure 6, each group contains about 10 authors.  Figure 6(a) shows the cooperation group of authors with the largest publications on conjunctival bacteria. There are 12 authors in this group with 46 papers, most of which were published in 2001-2010.  Figure 6(b) shows the cooperation group of authors with the second largest publications on conjunctival bacteria. There are 10 authors in this group with 24 papers, most of which were published in 1991-2019.  Figure 6(c) shows the cooperation group of authors with the third largest publications on conjunctival bacteria. There are 9 authors in this group with 22 papers, most of which were published in 1996-2019.

### 3.3. Discipline and Journal Analysis

**Figure 7:**
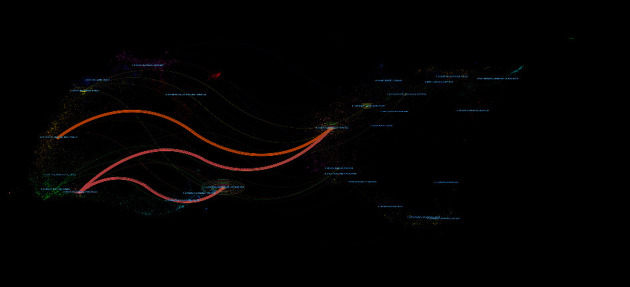
Dual-map overlays of discipline and journal analysis related to conjunctival bacteria. All the citing journals are on the left side, and all the cited journals are on the right side. The color of links distinguished the disciplinary of source.

The result of discipline and journal analysis related to conjunctival bacteria was shown in Figure 7, a dual-map created using CiteSpace. As shown in Figure 7, the referential links originate from a citing journal on the left side of the dual-map and point at a cited journal on the right side. The color of links distinguished the disciplinary of source. Overall, the citing journals that published conjunctival bacteria belong to mathematics, medical, ecology, molecular biology, physics, material, and chemistry, whereas the cited papers were published with a focus on molecular biology, genetics, nursing, medicine, surgery, parasitology, toxicology et al. Regarding the top 5 cited journals, *Ophthalmology* had the highest IF in 2018 (7.732) and contributed the most articles on conjunctival bacteria (135 articles). Other contributing journals include *American Journal of Ophthalmology* (119 articles), *British Journal of Ophthalmology* (113 articles), *Archives of Ophthalmology* (109 articles), *Investigative Ophthalmology*, and *Visual Science* (105 articles).

### 3.4. Cluster Analysis of Research Trends

In order to reveal the research trends and hotspots of conjunctival bacteria, we ran the Citesapce with the node type of “category”. The top 100% of slices were included.  Table 2 shows the categories with 10 publications or more that relate to conjunctival bacteria. According to the results, as the leading cause of conjunctivitis and ocular surface infection, most papers (142 of 285 publications, 49.82%) that relate to conjunctival bacteria fall into the category of ophthalmology. Other categories include veterinary sciences (53 of 285 publications, 18.60%), pharmacology and pharmacy (19 of 285 publications, 6.67%), microbiology (19 of 285 publications, 6.67%), and immunology (17 of 285 publications, 5.96%).

**Figure 8:**
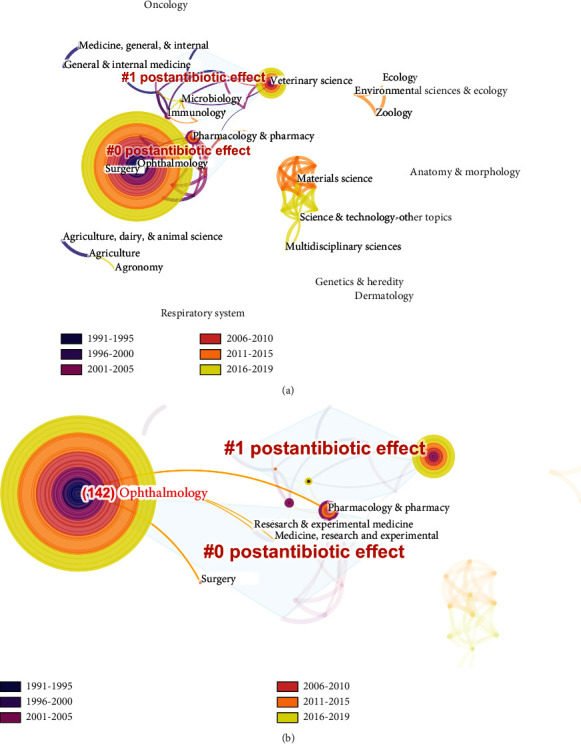
Categories map of published literature related to conjunctival bacteria. (a) All the 49 categories were connected with 89 links. (b) The category with the largest number of published literatures, ophthalmology, is linked to 4 categories.

The result was then organized into two same clusters (postantibiotic effect), as shown in Figure 8(a). There are 49 nodes connected with 89 links. As shown in Figure 8(b), there are 4 categories that link to ophthalmology, including pharmacology and pharmacy, research and experimental medicine, medicine, research and experimental, and surgery.

### 3.5. Burst Keywords Analysis of Hotspots

**Figure 9:**
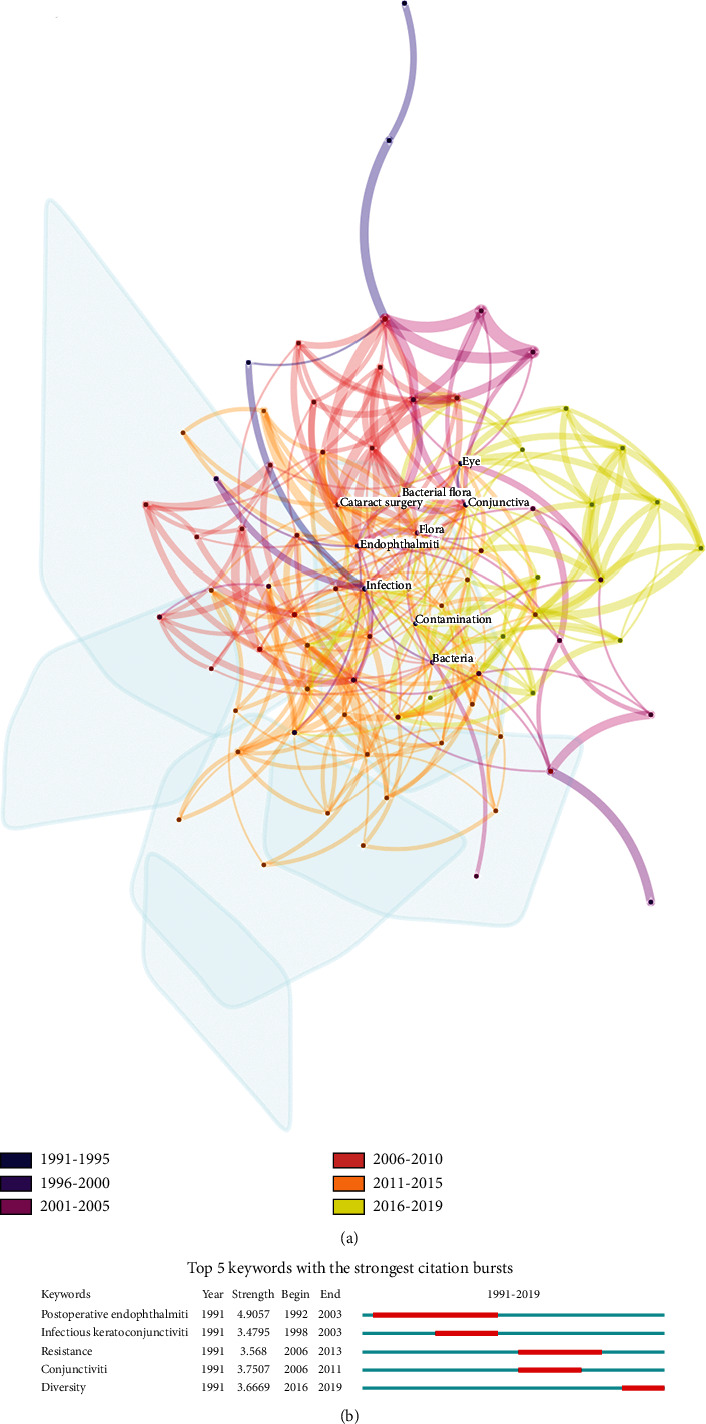
The keywords distribution and burst keywords analysis of conjunctival bacteria. (a) Cooccurrence network of keyword analysis of conjunctival bacteria. Color of the nodes showed the corresponding time period of different keywords. Link between 2 nodes reflects there is a cooccurrence relationship between the 2 keywords. (b) The top 5 keywords with the strongest citation bursts. Blue line represents the base timeline, and red part indicates the burst duration of each keyword.

Keywords can accurately reflect the research hotspots of the SCI literature at a certain time. Besides, identifying burst keywords among all the keywords may help to predict new frontier topics or research trends in the future [22]. Thus, in order to understand the development of conjunctival bacteria in a more comprehensive manner, we used CiteSpace to extract and sort out all the keywords in the literature related to conjunctival bacteria. There are 1521 extracted keywords, and Table 3 shows the keywords that are included in 10 conjunctival bacteria-related publications or more. To further screen keywords with high frequency and display the network in a more direct way, we chose the top 5.0% per slice and ran the software again. The keywords and the relationships among them were shown as 81 nodes and 353 links in Figure 9(a) and the top 5 keywords with the strongest citation bursts were shown as Figure 9(b). The keyword with the strongest citation strength was “postoperative endophthalmiti(s)” (4.9057), and this trend lasted for 11 consecutive years (1992-2003). The strongest cited keyword (“postoperative endophthalmiti(s)”) together with the other 2 strongly cited keywords, “infectious keratoconjunctiviti(s)” (3.4795,1998-2003) and “conjunctiviti(s)” (3.7507, 2006-2011), is the 3 common infectious diseases caused by conjunctival bacteria. Moreover, in order to decrease the incidence of bacterial infections, use of antibiotics has become an effective strategy. However, with the widespread use of antibiotics, the rate of antimicrobial resistance gradually increased. The keyword “resistance” (3.568) has become one of the strongest citation bursts since 2006, and the trend lasted for 7 years. To further identify and study the species of conjunctival bacteria of patients with different diseases, researches focus on the diversity of conjunctival bacteria. The keyword “diversity” (3.6669) is the latest burst keyword and began from 2016.

## 4. Discussion

Based on the research methods of bibliometric studies and characteristics of ophthalmology, our study explored the bibliometric characteristics of conjunctival bacteria research by analyzing the published literature of conjunctival bacteria from 1991 to 2019 using CiteSpace V (version 5.5R2). In total, we screened out 285 relevant papers, which was about 100 less than the publication numbers of conjunctival virus (420 relevant papers). Most of these papers were published in journals that belong to the categories of medical and molecular biology. Prior to 1990, the field of conjunctival bacteria has not attracted much attention of researchers. However, according to our study, the number of literatures on conjunctival bacteria has been increasing since then, especially in the past decade. At the same time, with the development of research on conjunctival bacteria, the citation frequency has generally increased over time, reaching the peak in 2017. This may be related to the increase of researchers' attention to the relationship between conjunctival bacteria and ocular infection.

By reviewing the global distribution map of the number of published literatures on conjunctival bacteria (Figure 3), we can easily conclude that the United States, Germany, Japan, China, and Italy contribute most to the publication of conjunctival bacteria. From the cooperation network map of countries/regions, institutions, and authors on conjunctival bacteria (Figures 4–6), we could see that there are several collaborating relationships among these countries and institutions. As the countries that have made important contributions in this field, the United States and Germany cooperate more often. The Stanford University (12 papers) is the most productive institution in the United States, while the University of Munich (12 papers) contributes most in the field of conjunctival bacteria in Germany. Most of their papers were published between 2001 and 2005, mainly involving ocular infection caused by conjunctival bacteria and the efficacy and side effects of different kinds of antibiotics [23–28]. These papers lay the foundation for later research of conjunctival bacteria. However, as we found, the cooperation among other institutions are not close. We speculate that the possibility of this phenomenon is that the research of conjunctival bacteria has not been paid enough attention. Another thing to note is that we could not distinguish the real contribution of different authors in complex cooperative relationship through the analysis of bibliometrics software. For instance, Nentwich et al. have been conducting research on the incidence of postoperative endophthalmitis caused by conjunctival bacteria in Germany [29]. Lalitha et al. studied on unbiased pathogen detection and host gene profiling for patients with conjunctivitis in India [30]. All the data of these two studies were analyzed in collaboration with Stanford University of the United States, and thus both the corresponding authors of these two papers were from the United States. Considering the effort of collecting information from participants, it is likely that the contribution of institutions in Germany and India may be underestimated. Thus, it requires researchers to read the original literature themselves to determine the authors that provide the greatest contribution. By this way, researchers can strengthen exchanges and cooperation between different institutions in this field.

Judging from the number of literatures, Christopher N Ta (12 literatures) and Herminia Mino de Kaspar (10 literatures) are in the first place and the second place among all authors, indicating that they are the most productive and influential scholar in this field. Their research mainly includes several parts, and the assessment of different antimicrobial agents on reducing conjunctival bacterial contamination rate is one of them. In 2002, Ta et al. first conducted a prospective randomized study on preoperative ofloxacin prophylaxis for cataract surgery and pointed out that the application of topical ofloxacin for 3 days before surgery appears to be more effective than the application of it 1 hour before surgery [31]. Then, a number of experiments have been carried out successively on prophylaxis therapy or treatment of ocular infection using different antimicrobial agents, including minocycline, ofloxacin, povidone-iodine, levofloxacin, moxifloxacin, and gatifloxacin [25, 29, 32–43]. Besides, with the increasing incidence rate of resistance towards multidrug, the research focus of researchers is gradually turning to drug resistance of conjunctival bacteria. By analyzing a large number of cases, they also summarize that patients with local and/or systemic risk factors are more likely to harbor multiresistant organisms and have a higher rate of bacterial conjunctival contamination before intraocular surgery [23, 44]. Also, their cooperation group have revealed that patients with intraocular infection after intravitreal injections are mostly infected by coagulase-negative staphylococci, which is sensitive to vancomycin, gentamicin, and chloramphenicol [45]. Due to the important role in clinical management of conjunctival bacterial infection, it is easy to explain why antimicrobial agents attracted so much attention. However, the mechanism of the invasion into ocular surface by different species of conjunctival bacteria has not been studied in depth or widely verified. Also, we notice that there are several complicated collaborating networks of authors of conjunctival bacteria in addition to Christopher and Herminia's cooperation group. Unfortunately, there is not enough contact or cooperation among them at present.

Keywords and topic clusters can serve as important index to reflect the research hotspots of the SCI literature at a certain time. As we found, the burst keywords and topic clusters in the field of conjunctival bacteria can be divided into several groups. “Diversity” (3.6669, bursted from 2016 to 2019) has become the latest burst keyword since 2016. With the development of conjunctival bacteria research, researchers found that the species and distribution of conjunctival bacteria were different in patients with different diseases. Even in normal people, different ocular surface environment may lead to different species and distribution of conjunctival bacteria. In 2016, Cintia S de Paiva et al. found that the severity of Sjögren syndrome ocular and systemic disease was inversely correlated with microbial diversity [46]. In 2018, Ham et al. identified a significant difference in the microbial community composition between diabetic patients and healthy subjects [47]. According to their study results, a high abundance of *Acinetobacter* in the ocular surface of diabetic patients may arise from the unique characteristics of the ocular surface compared with those of other organ surfaces. In 2019, Dong et al. found that patients with the meibomian gland dysfunction may have various degrees of imbalance of conjunctival bacteria. *Staphylococcus*, *Corynebacterium*, and *Sphingomonas* may play roles in the pathophysiology of the meibomian gland dysfunction [48]. From these studies, we can summarize that different diseases have different effects on conjunctival bacteria, thus leading to different composition and distribution of the flora. Thus, antibiotic therapy may be individualized. Considering the various impacts, studies on the diversity of conjunctival bacteria may become a research hotspot for a long time in the future.

With an eye on the common infectious diseases caused by conjunctival bacteria, “postoperative endophthalmiti(s)” (4.9057 bursted from 1992 to 2003), “infectious keratoconjunctiviti(s),” (3.4795 bursted from 1998 to 2003), and “conjunctiviti(s)” (3.7507 bursted from 2006 to 2011) are the three keywords detected through burst analysis. Among them, the keyword “postoperative endophthalmiti(s)” is the strongest cited keyword. Postoperative endophthalmitis is one of the most feared complications of intraocular surgery which is usually associated with impaired vision and may even lead to complete vision loss [49]. It always happens after the most common types of intraocular surgeries including cataract extraction trabeculectomy and pars plana vitrectomy (PPV). It was reported that the incidence of endophthalmitis after cataract surgeries were 0.012%-1.3% with improved instrumentation and small incision surgery [50]. As for trabeculectomy, early studies showed that the incidence rate of endophthalmitis ranged from 1.9% to 13% [51, 52]. For patients undergoing PPV surgeries, the incidence of postoperative endophthalmitis varies with different surgical equipment. According to early studies, the incidence ranged from 0.03% to 0.14% for 20G PPV, and it ranged from 0.23% to 0.84% for 25G PPV [53–55]. According to several studies, the risk factors include surgical factors and systemic factors. Major surgical factors are posterior capsular rupture poor corneal wound construction and other inadequate wound closure leading to hypotony [50, 56–60]. As for systemic risk factors, patients with diabetes mellitus are more likely to be infected after surgeries [50, 53, 58, 61]. As for microbiology, the most prevalent organisms of culture-positive postcataract endophthalmitis are the gram-positive organisms, and 70% of gram-positive bacteria are coagulase-negative *Staphylococcus* [62–64].

Antimicrobial agents are often used for clinical treatment and management of conjunctival bacteria. According to our bibliometric study, the group of application of antibiotics consists two keywords and topic clusters: “postantibiotic effect” and “resistance.” Postantibiotic effect refers to the temporary suppression of bacterial growth following transient antibiotic treatment, which has been observed for decades for a wide variety of antibiotics and microbial species [65]. It can effectively reflect the sensitivity and resistance of conjunctival bacteria towards antibiotics, and thus a large number of relevant studies have been conducted. According to their results, most of the detected conjunctival bacteria species, including coagulase-negative *Staphylococci*, *Staphylococcus aureus*, *Streptococci* spp., *Cutibacterium spp*., *Escherichia spp.*, and *Acinetobacter* spp., are sensitive to several antibiotics [5, 25, 29, 32–43, 66–74]. Among them, levofloxacin, a fluoroquinolone antibacterial agent, is widely used for treating common external infections and widely used in prophylaxis in patients undergoing ocular surgery [66]. Its good postantibiotic effect depends on high concentration in tears and the broad spectrum of activity against Gram positive and negative bacteria.

However, with the widespread use of antibiotics, the antimicrobial resistance rate gradually increased and thus left a negative impact on postantibiotic effect. The keyword “resistance” (3.568, bursted from 2006 to 2013) has become one of the strongest citation bursts since 2006, and the trend lasted for 7 years. In 2018, the Antibiotic Resistance Monitoring in Ocular Microorganisms (ARMOR) surveillance study evaluates in vitro antibiotic resistance among several conjunctival-sourced ocular isolates collected across the US from 2009 through 2016 [75]. According to the study, a large proportion of Staphylococci demonstrated resistance to oxacillin and azithromycin. Multidrug resistance (>3 antibiotic classes) was found in 30.2% of Staphylococcus aureus and 39.0% of coagulase-negative Staphylococci isolates. The widespread emergence of antibiotic-resistant pathogens has become a severe threat to public health. Considering the antimicrobial resistance in the current research field of conjunctival bacteria, it must be a research hotspot and requires more studies in the future.

The global field of conjunctival bacteria has expanded rapidly in the last 28 years. To our knowledge, this is the first bibliometric study to analyze this topic. However, a few limitations of bibliometric studies must be considered. First, based on the limited amount of literature, it is difficult to obtain enough and effective information to reveal the rule and predict the trend and hotspots of the research. Second, all of our data were retrieved from the Web of Science Core Collection database, and we cannot ensure all relevant studies were included. Third, bibliometrics software cannot distinguish the real contribution of different authors in complex cooperative relationship, but can only analyze the amount of literature provided by the authors in this field. Thus, it requires researchers to read the original literature themselves. Fourth, though the analysis is conducted objectively by software, there are inherent subjective biases in the way these results are interpreted.

Still, we believe that our analysis can reflect the general trend and hotspots of conjunctival bacteria research. Compared with the traditional reviews, CiteSpace-based analysis could visualize the data and provide better insight of the history, status, and focus of conjunctival bacteria research. The use of bibliometrics software could be considered as an auxiliary means to enter the field of research easily. Once the researchers have a preliminary understanding of the research field, they can further obtain their own judgment by studying the original manuscript.

## 5. Conclusions

The global field of conjunctival bacteria has expanded rapidly from 1991 to 2019. The United States contributes most and plays an important role in the network of cooperation between countries. However, there are little cooperation among authors and institutions. Overall, this bibliometric study organized one cluster, “postantibiotic effect”, and identified the top 5 hotspots in conjunctival bacteria research: “postoperative endophthalmiti(s),” “infectious keratoconjunctiviti(s),” “conjunctiviti(s),” “resistance,” and “diversity”. Thus, further research focuses on these topics may be more helpful to prevent ocular infection and improve prophylaxis strategies to bring a benefit to patients in the near future.

## Figures and Tables

**Table 1:** The top 10 countries/regions and top 11 institutions contributed to publications of conjunctival bacteria from 1991 to 2019.

Country/region (rank)	Record	Institution (rank)	Record
USA (1)	82	Univ Munich (1)	12
Germany (2)	23	Stanford Univ (1)	12
Japan (2)	23	Univ Catania (3)	5
China (4)	18	Islamic Azad Univ (3)	5
Italy (4)	18	Osaka Univ (3)	5
England (6)	17	Univ Messina (6)	4
India (7)	12	Kansas State Univ (6)	4
Turkey (8)	11	Baylor Coll Med (6)	4
Australia (8)	11	Natl Vet Inst (6)	4
Brazil (8)	11	Harvard Med Sch (6)	4
		Univ New S Wales (6)	4

**Table 2:** The categories with 10 publications or more that relate to conjunctival bacteria.

Category	Record	Percent of 285
Ophthalmology	142	49.82%
Veterinary sciences	53	18.60%
Pharmacology and pharmacy	19	6.67%
Microbiology	19	6.67%
Immunology	17	5.96%
Infectious diseases	15	5.26%
Surgery	10	3.51%
Medicine, General, and internal	10	3.51%
General and internal medicine	10	3.51%

**Table 3:** The keywords that are included in 10 conjunctival bacteria-related publications or more.

Keyword	Record	Percent of 285
Conjunctiva	59	20.70%
Bacteria	47	16.49%
Eye	35	12.28%
Infection	33	11.58%
Endophthalmiti	32	11.23%
Keratiti	27	9.47%
Postoperative endophthalmiti	23	8.07%
Flora	23	8.07%
Conjunctiviti	22	7.72%
Cataract surgery	20	7.02%
Bacterial flora	20	7.02%
Povidone iodine	17	5.96%
Prophylaxi	16	5.61%
Identification	15	5.26%
Resistance	15	5.26%
Prevalence	15	5.26%

## Data Availability

The datasets used and analyzed during the current study are available from the corresponding author on reasonable request.
